# Supplementary figures and images for: Variations in gut bacterial communities of hooded crane (Grus monacha) over spatial-temporal scales
Source: PeerJ. 2019 Jun 10;7:e7045. doi: 10.7717/peerj.7045 (PMC6563796; doi:10.7717/peerj.7045)

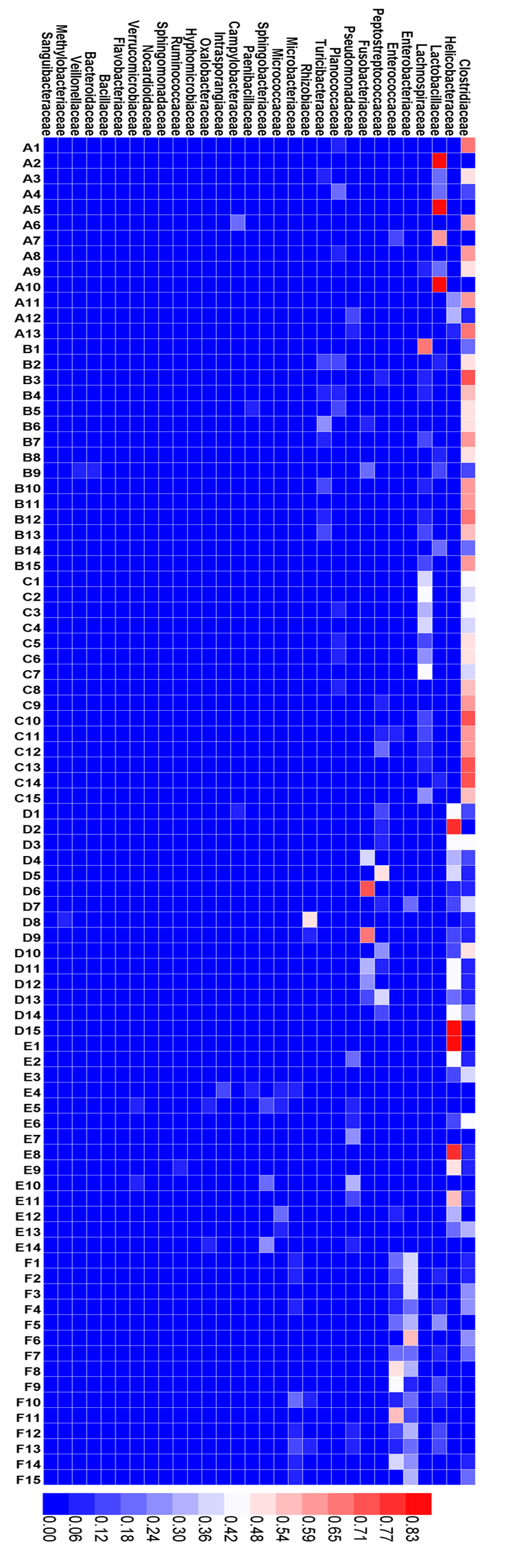

Supplement: Figure S1 — Heatmap showing the relative abundance at the family level. [file peerj-07-7045-s002.png]

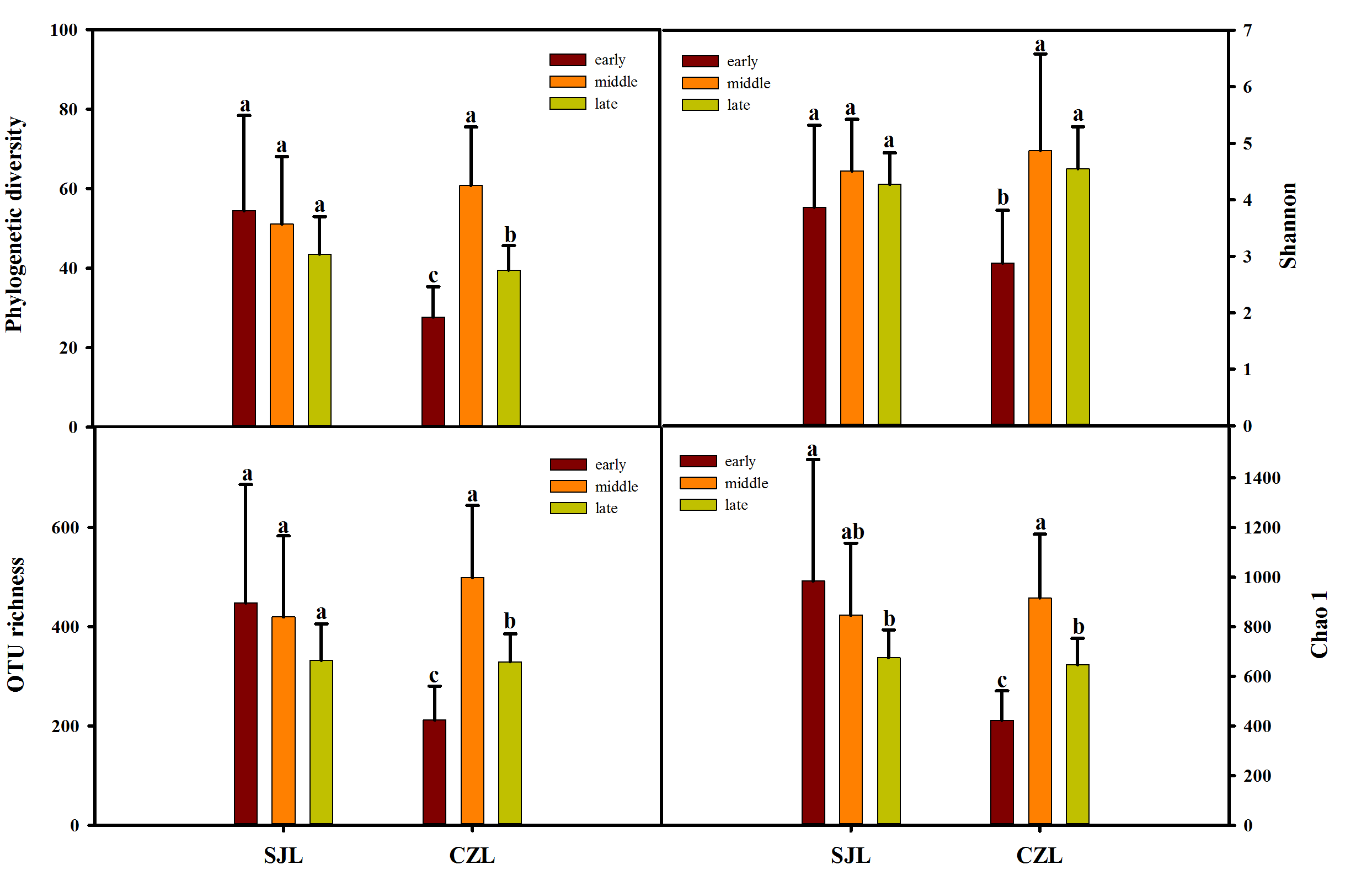

Supplement: Figure S2 — Variations in diversity (Phylogenetic diversity, OTU richness, Chao 1 and Shannon) in different sampling location. Different letters represent significant differences by One-way ANOVA ( P < 0.05). Error bars indicate standard deviation. [file peerj-07-7045-s003.png]

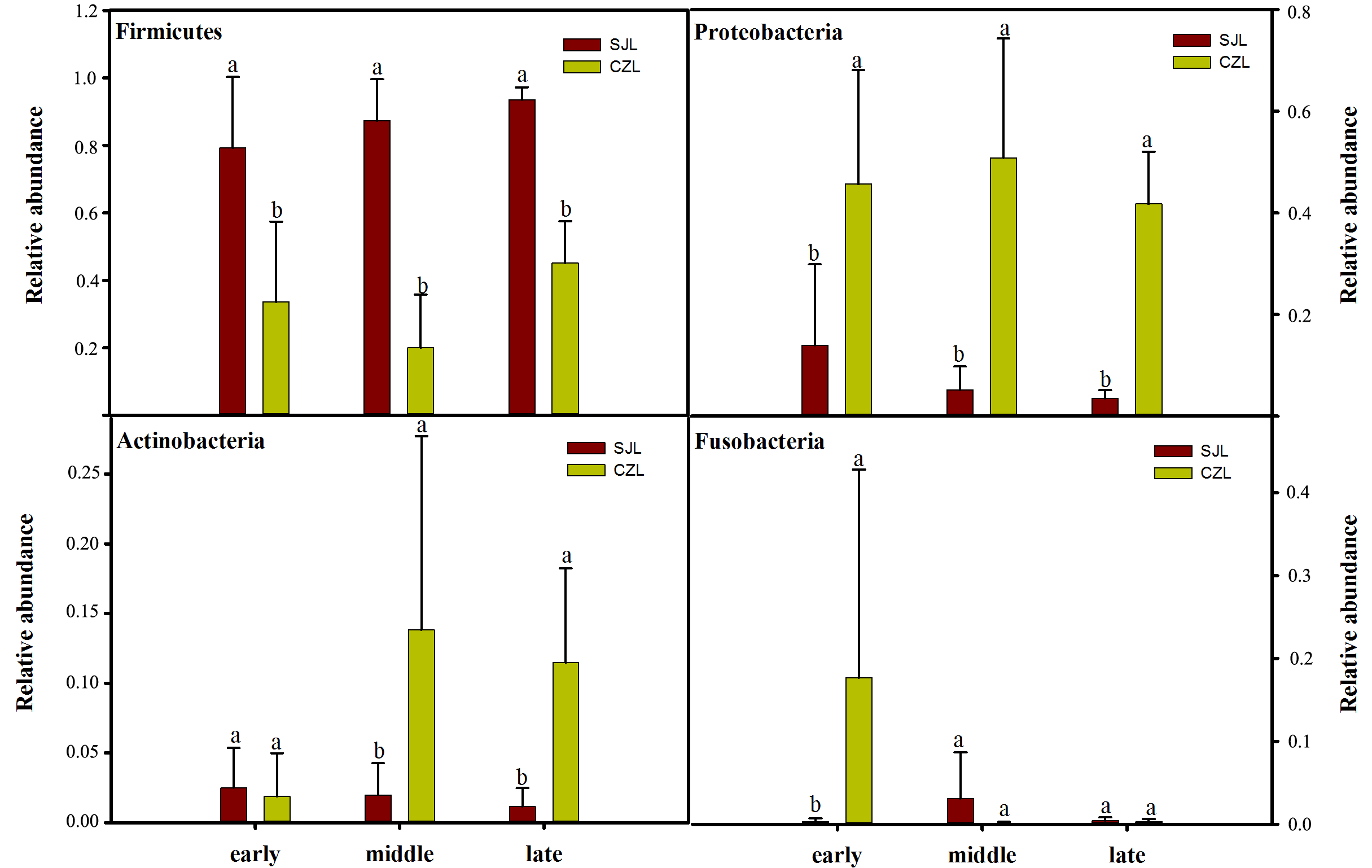

Supplement: Figure S3 — Different letters represent significant differences by One-way ANOVA (P < 0.05). Error bars indicate standard deviation. [file peerj-07-7045-s004.png]

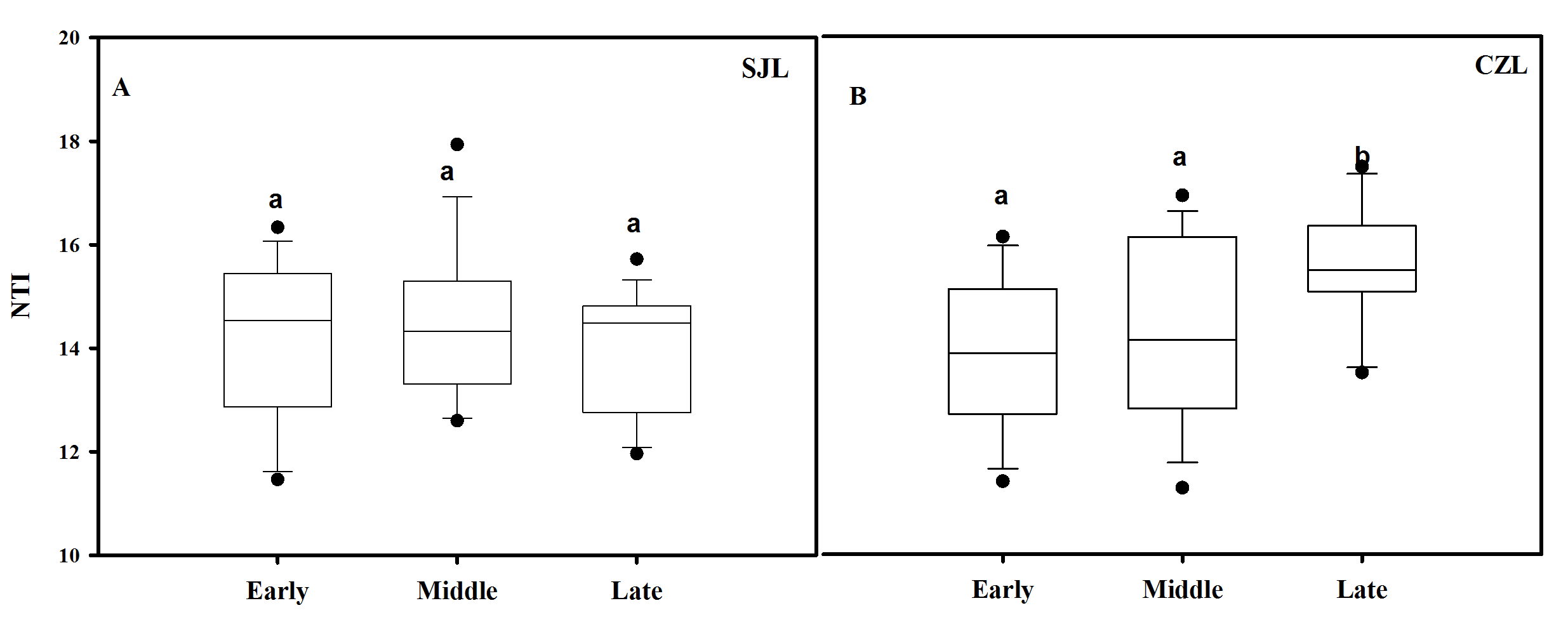

Supplement: Figure S4 — Bacterial community phylogenetic structure evaluated by the NTI in different sampling location. (A) In the Shengjin Lake. (B) In the Caizi Lake. [file peerj-07-7045-s005.png]
