# Supplementary material for: Variations in gut bacterial communities of hooded crane (Grus monacha) over spatial-temporal scales
Source: PeerJ. 2019 Jun 10;7:e7045. doi: 10.7717/peerj.7045 (PMC6563796; doi:10.7717/peerj.7045)
Supplement: Table S1 — Differences in the microbial community composition based on the Two-way ANOVA (P < 0.05). [file peerj-07-7045-s006.docx]

**Table S1**. Variations in diversity (Phylogenetic diversity, OTU richness, Chao 1 and Shannon) in different sampling location. Different letters represent significant differences by Two-way ANOVA (*P* < 0.05).

|  | temproal change | | spatial change | | temproal*spatial | |
| --- | --- | --- | --- | --- | --- | --- |
|  | F | *P* | F | *P* | F | *P* |
| PD | 10.296 | 0.000 | 5.298 | 0.024 | 11.969 | 0.000 |
| chao1 | 5.922 | 0.004 | 9.694 | 0.003 | 12.136 | 0.000 |
| observed species | 8.588 | 0.000 | 3.256 | 0.075 | 10.124 | 0.000 |
| shannon | 11.196 | 0.000 | 0.232 | 0.631 | 3.321 | 0.041 |
