# Supplementary material for: Variations in gut bacterial communities of hooded crane (Grus monacha) over spatial-temporal scales
Source: PeerJ. 2019 Jun 10;7:e7045. doi: 10.7717/peerj.7045 (PMC6563796; doi:10.7717/peerj.7045)
Supplement: Table S2 — Summary of key findings in differences between predicted metagenomes . [file peerj-07-7045-s007.docx]

**Table S2.** Summary of key findings in differences between predicted metagenomes.

| Predicted Functional | Sample 1 | Propotion of metagenome (%) | Sample 2 | Propotion of metagenome (%) | *p* value |
| --- | --- | --- | --- | --- | --- |
| Metabolic Diseases | SJL-E | 11.30 | CZ-E | 12.63 | 0.034 |
|  | SJL-M | 12.38 | CZ-M | 11.01 | 0.003 |
|  | SJL-L | 12.90 | CZ-L | 16.46 | <0.001 |
| Carbohydrate Metabolism | SJL-E | 8.46 | CZ-E | 9.19 | 0.002 |
|  | SJL-M | 8.93 | CZ-M | 8.82 | 0.655 |
|  | SJL-L | 9.10 | CZ-L | 10.98 | <0.001 |
| Amino Acid Metabolism | SJL-E | 8.57 | CZ-E | 9.36 | <0.001 |
|  | SJL-M | 8.75 | CZ-M | 9.52 | <0.001 |
|  | SJL-L | 8.89 | CZ-L | 9.70 | <0.001 |
| Sensory System | SJL-E | 8.25 | CZ-E | 8.34 | 0.708 |
|  | SJL-M | 8.03 | CZ-M | 9.47 | <0.001 |
|  | SJL-L | 8.14 | CZ-L | 7.08 | <0.001 |
| Transport and Catabolism | SJL-E | 5.34 | CZ-E | 5.57 | 0.290 |
|  | SJL-M | 5.07 | CZ-M | 6.37 | <0.001 |
|  | SJL-L | 5.14 | CZ-L | 4.20 | <0.001 |
| Replication and Repair | SJL-E | 4.90 | CZ-E | 4.64 | <0.001 |
|  | SJL-M | 4.99 | CZ-M | 4.48 | <0.001 |
|  | SJL-L | 5.00 | CZ-L | 5.12 | 0.004 |
| Circulatory System | SJL-E | 3.60 | CZ-E | 3.85 | 0.042 |
|  | SJL-M | 3.73 | CZ-M | 3.48 | 0.021 |
|  | SJL-L | 3.93 | CZ-L | 3.77 | 0.220 |
| Membrane Transport | SJL-E | 2.74 | CZ-E | 2.78 | 0.582 |
|  | SJL-M | 2.77 | CZ-M | 3.12 | 0.002 |
|  | SJL-L | 2.77 | CZ-L | 3.07 | <0.001 |
| Glycan Biosynthesis and Metabolism | SJL-E | 3.04 | CZ-E | 3.02 | 0.909 |
|  | SJL-M | 2.93 | CZ-M | 2.68 | 0.027 |
|  | SJL-L | 2.84 | CZ-L | 2.26 | <0.001 |
| Genetic Information Processing | SJL-E | 2.92 | CZ-E | 2.62 | 0.010 |
|  | SJL-M | 2.65 | CZ-M | 3.02 | 0.003 |
|  | SJL-L | 2.53 | CZ-L | 2.17 | <0.001 |
| Metabolism of Cofactors and Vitamins | SJL-E | 2.60 | CZ-E | 2.53 | 0.320 |
|  | SJL-M | 2.72 | CZ-M | 2.22 | <0.001 |
|  | SJL-L | 2.78 | CZ-L | 2.73 | 0.262 |
| Cellular Processes and Signaling | SJL-E | 2.06 | CZ-E | 3.46 | <0.001 |
|  | SJL-M | 2.08 | CZ-M | 2.62 | 0.078 |
|  | SJL-L | 2.36 | CZ-L | 1.99 | 0.010 |
